# Computer-based analysis of verbal autopsies: revolution or evolution?

**DOI:** 10.1186/1478-7954-9-26

**Published:** 2011-08-01

**Authors:** Ian Riley

**Affiliations:** 1 School of Population Health, University of Queensland, Brisbane, Australia

## Scientific revolution and the clinico-pathological paradigm

In this edition of *Population Health Metrics*, a series of papers describes new, automated methods for analyzing verbal autopsy questionnaires. Are we witnessing a revolution in the computer-based analysis of verbal autopsies? The use of the word, *revolution*, brings to mind Thomas Kuhn's seminal essay, *The Structure of Scientific Revolutions*, first published in 1962, and his concept of the scientific paradigm [1].

Verbal autopsies, as we now know them, are not a new concept, but rather are late developments within the clinico-pathological paradigm that displaced the humoral theory of disease in the late 18^th ^and early 19^th ^centuries. We could mark the beginnings of this scientific revolution from the publication in 1761 of Morgagni's *The Seats and Causes of Disease Investigated by Anatomy *[2]. Encyclopedic in scope, it is over 2,000 pages in length in its English translation. His general plan is to present symptom histories of patients who had died, describe the findings at autopsy, speculate on the likely causal relationships between pathology and symptoms, and finally to discuss similar cases found in a literature extending back from recent centuries to the ancients. The first level of classification is by the three major body cavities (head, thorax, and belly [sic]) and surgery; the second level is by symptom group. At the end of the three volumes are his indices, which he regarded as critical: one of these lists symptoms alphabetically, cross-referencing them to the pathology of disease case-by-case; another lists pathological lesions, similarly cross-referencing them to symptom histories. His underlying thesis, that clinical symptoms reflect organ dysfunction, was not to be fully accepted for over half a century. This thesis can be briefly stated, but it is the sheer weight of evidence, somewhat in the manner of *The Origin of Species*, that makes his case. That was not his only purpose: he wanted this to be a working manual (albeit a very large one) for physicians and anatomists.

Equally, we could mark the endpoint of this scientific revolution with the publication in 1819 of another massive work - Laennec's *A Treatise on Diseases of the Chest *[3]. Laennec argued that the symptom history was inaccurate. Using his new invention, the stethoscope, he linked detailed auscultatory findings to autopsy findings of pulmonary pathology. All of modern medicine rests upon this linkage. Morgagni validated pathological anatomy as the cause of disease by using the symptom history as his gold standard. Sixty years later, Laennec dismissed the symptom history as inaccurate and validated auscultation using pathological anatomy as his gold standard. In the course of this paradigm shift, pathology had been moved from the margins of the medical solar system to its heart. In Foucault's words, "The space of configuration of the disease" was now superimposed "upon the space of the localization of illness." [4]

Medical practice in the 18^th ^century had depended heavily on what we would now call unstructured symptom histories. Physical examination was limited in the main to the observation of the fully clothed patient and palpation of the pulse. Laennec's work led to a wave of enthusiasm for auscultation to the exclusion of other methods of diagnosis. In attempting to restore balance, Pierre Louis "initiated a method of clinical teaching based on precise observation and statistical analysis." He began the structuring of the clinical history by introducing direct questioning. In the words of Stanley Reiser [5]:

Louis objectified each footprint of disease by numeration. For him all signs had equal merit, the criteria for their excellence being the care with which they were observed and described, and their statistical correlation with a particular disease, checked when possible by autopsy.

If we wanted a direct ancestor for our work on verbal autopsies, we might do worse than by adopting Pierre Louis for whom it was "indispensable to count." [6]

By the middle of the 19^th ^century, the new paradigm had won general acceptance. From then on we can think of clinical diagnosis in terms of its three pillars: clinical history, physical examination, and laboratory investigation. Its current form as a semistructured interview was established in the early 20^th ^century. The formal physical examination with its successive steps of observation, palpation, percussion, and auscultation, too, was fixed comparatively early.

The great scientific advances lay in the development of new branches of pathology - histopathology, pathophysiology, microbiology, clinical biochemistry, and medical genetics - and in imaging. New methods of investigation gave the physician increasingly direct access to the pathology of the living body. Autopsies became rarities and the pathological anatomy of Morgagni and his successors was relegated to bottles in museums.

## The verbal autopsy: clinical parallels

The lineage of the verbal autopsy instrument should now be clear. It was based on the clinical history and, like the clinical history, passed through a phase of unstructured narrative before being adapted as a structured survey instrument. Verbal autopsy diagnostics then passed through phases of physician review, of Bayesian analysis based on prior probabilities, and most recently of machine learning. An estimate based on machine learning is that the verbal autopsy is 75% accurate when measured against clinical gold standards, well exceeding human accuracy in the analysis of the same instrument [7].

The reactions of physicians to this demonstration of the power of machine learning to realize the information content of the autopsy is reminiscent of the reactions of chess players on hearing of Garry Kasparov's defeat by the IBM supercomputer, Deep Blue, in 1997: one senses feelings not only of chagrin but also of regret that yet another domain of what had appeared to be peculiarly human reasoning had yielded to the power of computers. It would be appropriate to ask, therefore, exactly how physicians do reason when they make clinical diagnoses.

This question assumed increasing importance for medical educators with the introduction of curricula based on problem-solving. Hitherto the emphasis had been on the accumulation of factual knowledge allied to bedside experience in hospital wards. The challenge they faced was whether diagnostic skills could be taught as such.

Initially, diagnosis was regarded as a linear process referred to as hypothetico-deductive reasoning, which involved successive steps of problem definition, formulation of tentative hypotheses, collection of preliminary data, formulation of a specific hypothesis, accumulation of further data that tested the hypothesis, and drawing of diagnostic conclusions [8]. It became obvious, however, that this was a weak process used principally by novices and not by expert diagnosticians. A second method, pattern recognition, used by experts working within a familiar field, was associated with 10-fold greater odds of diagnostic success in a test situation than was hypothetico-deductive reasoning. Aptitude depended on both an extensive knowledge base and extensive experience. The development of the necessary skills entailed a much more complex cognitive process than the name implies, and it could not be taught to novices. A third method, known as scheme-inductive reasoning, was associated with five-fold greater odds of diagnostic success. This method was used by experts in less-familiar situations and was also suitable for novice learners. Schemes reflect the organized structure of knowledge. They can be drawn on paper like inductive trees to recreate the major divisions, or chunks of information, used by expert clinicians for both storage and retrieval of knowledge in memory. Decisions are made explicitly at branches of the tree. "After several branching points, when the number of diagnostic options has been considerably reduced, deductive reasoning or pattern recognition may be exploited. Finally, the scheme-inductive process is not content-independent; each of the organizational schemes is specific to the clinical presentation." [9]

It is a rewarding intellectual experience for a clinician to work his or her way to an accurate diagnosis. Awareness of this, allied to pride in performance, will have colored physicians' reactions to learning of the superiority of machine learning over expert judgement. The suggestion that analysis of verbal autopsies is qualitatively different from other epidemiological analyses would seem to reflect intuitive awareness of the underlying complexity of cognitive processes. Large differences in diagnostic success rates between physicians and in one physician at different times is most probably a consequence of the large differences in success rates for different cognitive processes.

Criticisms of the clinical history include it being overly time-consuming, unreliable and, because of its subjectivity, unscientific. It certainly would not be regarded as the main platform for diagnosis. Yet a study in medical referral clinics in England, again by educationists, demonstrated that over 80% of diagnoses could be made accurately on the basis of general practitioners' referral letters and medical history [10]. The remainder of patients required physical examination and/or laboratory investigation to be made. These results need to be treated with caution but, given that a number of referral letters described symptoms alone and many patients would have been referred because they presented diagnostic difficulty, it seems reasonable to equate these results with the accuracy of verbal autopsy diagnoses based on a combination of symptom history and medical record recall by families. In short, for purposes of diagnosis the information content of the clinical history is much greater than many clinicians would be prepared to acknowledge.

Kuhn wrote about the transformation of a world view and of the "conversion" of scientists to a new paradigm [1]. Paradigm shift in Kuhn's terms was not of particular interest to Foucault [4]. He was concerned with the evolution of perception, language, and discourse, with their interdependence, and with the emergence of clinical science from the medicine of the 18^th ^century. One of his important arguments is that the disease of the 19^th ^century could not have been realized by the discourse of the 18^th^. To understand the processes of transformation of the medical worldview one should turn to Foucault. Considering his reputation as one of the architects of post-modernism, any doubts about the significance of the clinico-pathological paradigm to Western thought should be dispelled by this judgement [4]:

This structure in which space, death, and language are articulated - what is known, in fact, as the anatomo-clinical method - constitutes the historical condition of a medicine that is given and accepted as positive. Positive here should be taken in the strong sense. Disease breaks away from the metaphysic of evil, to which it had been related for centuries; and finds in the visibility of death the full form in which its content appears in positive terms.

It will no doubt remain a decisive fact about our culture that its first scientific discourse concerning the individual had to pass through this stage of death. ... from the integration of death into medical thought is born a medicine that is given as a science of the individual.

The structure of this commentary owes much to Reiser's *Medicine and the Rise of Technology *and his clear understanding of how overdependence on technology has distanced the physician from the patient as person. Given the above comments from Foucault, it is ironic that Reiser summarized observations by Kubler-Ross in these terms [11]:

... an impersonal clinical attitude and a wall of technology were ways of shielding medical personnel from anxiety-producing thoughts prompted by the critically ill or dying person, about their limitations and failures as healers, and their own mortality.

However, if prejudice against machine-based diagnosis rests on similar attitudes, it is misplaced. The arguments against continuing with physician-based certification of cause of death from verbal autopsies are too compelling: they relate to unreliability of diagnosis and the difficulties of maintaining quality work over long periods of time. The human interaction is between trained interviewers and families. If we have neglected the ethics of verbal autopsies then it is because we have not focused sufficiently on this interaction.

As physicians and epidemiologists, we work within the clinico-pathological paradigm without acknowledging it for what it is. We regard a way of thinking that links signs, symptoms, disease, and death as "natural" when, in fact, it is highly derived. It is odd that any high school science student is likely to be familiar with the work of Newton and Darwin but many health professionals would be pressed to give a coherent account of the great paradigm shift between the 18^th ^and 19^th ^centuries.

This affects our judgements about ourselves. The use of machine learning in assigning causes to verbal autopsies is likely to be revolutionary in terms of its impact in assigning causes to deaths outside hospitals worldwide, but the scientific revolution took place long before our time. "If we have seen further it is by standing on the shoulders of giants," [12] is a familiar saying but one containing much truth.

## Competing interests

The authors declare that they have no competing interests.

## Author's information

The author chaired the penultimate session on *Future Directions *at the Global Congress on Verbal Autopsy, Feb. 17, 2011.
